# A computational outlook on neurostimulation

**DOI:** 10.1186/s42234-020-00047-3

**Published:** 2020-05-25

**Authors:** Marco Capogrosso, Scott F. Lempka

**Affiliations:** 1 Department of Neurological Surgery, University of Pittsburgh, Pittsburgh, PA USA; 2 Rehabilitation Neural Engineering Laboratories, University of Pittsburgh, Pittsburgh, PA USA; 3 Department of Biomedical Engineering, University of Michigan, Ann Arbor, MI USA; 4 Biointerfaces Institute, University of Michigan, Ann Arbor, MI USA; 5 Department of Anesthesiology, University of Michigan, Ann Arbor, MI USA

**Keywords:** Neurostimulation, Neuromodulation, Computational modelling, Finite element modelling, Spinal cord stimulation, Chronic pain, Spinal cord injury

## Abstract

Efficient identification of effective neurostimulation strategies is critical due to the growing number of clinical applications and the increasing complexity of the corresponding technology. In consequence, investigators are encouraged to accelerate translational research of neurostimulation technologies and move quickly to clinical applications. However, this process is hampered by rigorous, but necessary, regulations and lack of a mechanistic understanding of the interactions between electric fields and neural circuits. Here we discuss how computational models have influenced the field of neurostimulation for pain and movement recovery, deep brain stimulation, and even device regulations. Finally, we propose our vision on how computational models will be key to accelerate clinical developments through mechanistic understanding.

## Background

In this perspective article, we sought to provide our personal experience and thoughts on the impact of computational models in the field of neurostimulation. We describe the general framework of technology development in the neurostimulation industry and provide examples of past, present, and potential future utility of computational models in accelerating technology development. We believe that our interpretation of the recent advancements in the field could help motivate other investigators to invest in the use of computational models, hopefully leading to a more precise interpretation of pre-clinical and clinical results.

## Main text

In the age of fast information transfer and social media, we are getting used to direct access to information and technology, on demand. We are convinced that this new interconnected environment allows inspiring ideas to quickly spin-off into high-profit, fast-success, high-tech solutions to environmental, social, and healthcare challenges of modern societies. However, this is not quite the pace of scientific discoveries. Scientific advances occur through a slow, laborious, and rigorous process requiring multiple experimental verifications and cross-validation procedures. This process is particularly true for biomedical applications in which significant costs and strict, but necessary, regulatory constraints bind the technology development to an even slower pace.

Despite this fact, the scientific community, and in particular the neuroscience community, is too quickly focusing on “translational applications” (i.e. the translation of scientific discoveries in neuroscience to clinical settings). Fostered by the urge to solve the impelling needs of an aging society, funding bodies provide ever-increasing support to this type of research. Given the stakes, as members of the scientific community and information-era human beings, we should question the very concept of translation, and approach this task with the most rigorous scientific attitude (Arber and Arber 2016).

Neuromodulation, or neurostimulation, technologies offer a clear example of this frantic race to clinical implementation. Both the scientific and industry communities seek new tools to interact with the nervous system and its computational architecture, without having a clear understanding of its particular features. Therefore, when developing neuromodulation technologies, engineers are asked to design devices that interact with largely undetermined systems, sometimes without having identified the actual neural targets. Verification of such systems is then sought in the preliminary outcomes of exploratory pilot clinical studies. However, given the titanic efforts and costs of clinical research, this purely experimental evidence-based approach is sub-optimal. Therapy optimization would be more efficient if at least part of the system efficacy was verified prior to finalization of the design. This initial verification would help focus design efforts on specific features, thus reducing the number and risks of experimental trials needed to refine therapies.

Computational models are natural candidates to perform this initial testing. The synthesis of state-of-the-art neuroscientific concepts into in-silico models of the nervous system simultaneously serves two purposes. First, it highlights how much we know of a specific system and where we should direct experimental research to acquire new knowledge (Markram et al. 2015). Second, it provides a virtual testing platform to study the interactions between neuromodulation technologies and the computational structure of the nervous system (McIntyre and Foutz 2013). After all, the efficacy of neuromodulation is determined by our ability to modify the outcome of the mathematical operations performed by complex networks of neurons. We can simulate these operations by implementing artificial representations of networks and their interactions with neuromodulation technologies.

We and others in the field have applied this strategy to characterize the interactions between spinal cord stimulation (SCS) and the dynamics of spinal circuits for the design of neuromodulation protocols to reduce chronic pain and to improve motor control in people with spinal cord injury. We believe that our personal experience in the use of computational models might provide a helpful example of the role that models could have in addressing important clinical and scientific questions. Ultimately, we are convinced that sharing our experiences and thoughts could help other investigators use this approach and help develop more effective therapies.

### Models of neurostimulation for the treatment of chronic pain

SCS for pain control is one of the most widely used forms of clinical neuromodulation. SCS for pain control was also one of the first applications of computational modelling to study the bioelectric effects of a clinical neuromodulation therapy. This rich history originally dates back to Sin and Coburn in the 1980s (Coburn 1985; Coburn and Sin 1985), expanded by Holsheimer and colleagues in the 1990s (Holsheimer 2002), and continues to this day. These seminal modelling studies not only provided insight into the direct neural response and potential mechanisms of action of SCS, but they also led to dramatic improvements in lead designs, stimulation configurations, waveform parameters, and programming procedures (Lempka and Patil 2018).

Although clinical demand has continued to increase over the years, technological and scientific developments in the SCS space remained stagnant for many years. However, over the last 5 years we have seen several new SCS technologies obtain CE marking and marketing approval by the United States Food and Drug Administration (FDA). Because several new devices have entered the clinical landscape, we now have increased competition that has forced companies to try and gain a competitive advantage. This competition may now prompt companies to rely more heavily on basic scientific discovery than in the past.

Although this increased competition may help advance the science of SCS for pain control, we need to be cognizant of the difference between scientific understanding and marketing strategies. For example, it is often presented that certain SCS waveforms and stimulation configurations can target the dorsal white matter tracts while others can directly target the neural elements within the gray matter of the spinal cord (Li et al. 2019). It is also argued that the specific features of bursting waveform patterns (e.g. passive versus active discharging, number of pulses) are critical to the overall stimulation efficacy (Kent et al. 2020; Meuwissen et al. 2018). Finally, another hypothesis is that the charge density or total amount of charge injected per unit time is a main determinant of SCS efficacy (largely independent of waveform shape) (Miller et al. 2016). While these concepts are exciting, additional work is necessary to investigate their validity. To help wade through the muddy and confusing waters of the neurostimulation space, we are often asked to speak to pain management physicians and scientists regarding what we actually know and don’t know about the science of SCS.

Unfortunately, SCS for pain control is an example of a therapy in which the cart has been put before the horse, i.e. clinical use and technological innovations, such as novel waveforms, advanced stimulator capabilities and lead designs, have outpaced our scientific understanding. From a clinical perspective, we still do not have a clear understanding of why SCS works well in some patients but fails in others. Due to challenges associated with objectively measuring pain, we are still restricted to assessing therapy efficacy with pain ratings and other outcome measures that are largely subjective in nature (Sankarasubramanian et al. 2019). From a scientific perspective, we still do not sufficiently understand the mechanisms of SCS-induced analgesia and if there are distinct mechanisms of action for specific types of SCS (e.g. tonic, burst, kilohertz-frequency) or for specific pain conditions. Furthermore, we are still learning how various anatomical factors affect the electric fields generated by SCS and how these electric fields translate into physiological and perceptual effects.

To improve scientific understanding of SCS and truly optimize these technologies, it is critical to perform systematic, well-powered, randomized, double-blind, placebo-controlled preclinical and clinical studies. In the past, it was challenging to perform placebo-controlled studies due to the paresthesias that were elicited by conventional SCS. However, several new forms of SCS, such as kilohertz-frequency and burst SCS, are applied at amplitudes below the patient’s sensory threshold and are therefore well suited for placebo-controlled studies. In parallel, we must continue efforts to develop patient-specific approaches investigating the physiological effects of SCS. Patient-specific computational models that account for various sources of interpatient variability, such as anatomy and electrode locations, are commonly used in other neurostimulation therapies to investigate mechanisms of action and provide clinical decision support (McIntyre and Foutz 2013). We firmly believe that patient-specific computational models of SCS (Lempka et al. 2019) will be critical to explain mixed outcomes in clinical trials of SCS and to support optimization of current and novel neurostimulation technologies.

### Models of neurostimulation of the spinal cord for motor control

Besides application in pain control, epidural neurostimulation of the spinal cord has shown the ability to improve motor control in animals (Capogrosso et al. 2018b; Courtine et al. 2009; Ichiyama et al. 2005) and humans with severe paralysis (Angeli et al. 2018; Gill et al. 2018; Harkema et al. 2011). Empirical observations provided evidence that during continuous electrical stimulation, spinal circuits in the rat spinal cord exploit peripheral sensory information to produce coordinated movement in the absence of supra-spinal inputs (Edgerton et al. 2008). These studies were a fascinating and clear example of how the behaviour of neural circuits could be modified by human intervention. Understanding how electrical stimulation is processed by the spinal circuits and transformed into coordinated locomotion is of critical importance for the translation of these promising results into viable clinical technologies.

We and others approached this problem by coupling electromagnetic models (Coburn 1985; Ladenbauer et al. 2010; Rattay 1986), similar to those developed for pain control, to classic experimental electrophysiology (Gerasimenko et al. 2006). We used this mix of computational models and model-driven experiments to verify hypotheses concerning the identification of the neural targets of epidural stimulation for motor applications. These experiments confirmed that neuromodulation at the lumbar spinal levels mainly recruits large sensory afferents (Capogrosso et al. 2013, 2018a) without direct recruitment of cells in the grey matter.

However, if peripheral sensory afferents are simultaneously the control signals (as hypothesized by Edgerton (Edgerton et al. 2008)) and the targets of neuromodulation, then why doesn’t this interaction interfere with the transmission of relevant information to the spinal circuits for the production of coordinated movements? In other words, how is neuromodulation altering the inputs to the computational machinery in the spinal cord? Given the even greater importance that sensory feedback has in the generation and control of human bi-pedal locomotion, this is far from being just a theoretical problem, potentially having important implications for the translation of these stimulation protocols to humans.

We posited that an artificial representation of the complex network controlling locomotion, or at least its first input layer, could help explain these interactions. Together with the team of Grégoire Courtine, we developed a simplified representation of the network responsible for the alternate recruitment of agonist and antagonist muscles in locomotion. We then coupled this simple network model to biomechanical models of the rat and human limbs, which provided realistic estimations of sensory inputs during gait (Formento et al. 2018; Moraud et al. 2016).

This modelling infrastructure provided a virtual environment to study the effects of neuromodulation during normal circuit dynamics. Using this model, we found that the elicitation of antidromic action potentials in the sensory afferents was disrupting natural information travelling orthodromically in these fibers during the gait cycle in humans, but not in rats. In rats, natural and stimulation-induced firing rates were integrated in a way that the balance between flexor and extensor muscle stretch information was preserved. Stimulation simultaneously increased excitation in motoneurons and other cells via natural synaptic terminals. However, the situation was more complicated in humans. If stimulation was delivered at supra-threshold levels, continuous electrical stimulation of the sensory afferents would cancel natural proprioceptive information. This cancellation was due to specific physical properties of the human anatomy. Specifically, fiber lengths and action potential transmission velocity in humans account for a natural sensory signal transmission latency to the spinal circuits on the order of 10 to 20 ms. These latencies, coupled to typical stimulation frequencies of 30 to 100 Hz (Harkema et al. 2011; Wagner et al. 2018) and human proprioceptive firing rates (likely < 100 Hz) (Formento et al. 2018), generate a situation in which complete cancellation of natural sensory information can occur during continuous stimulation at frequencies ≥40 Hz. Indeed, people implanted with epidural leads failed to perceive passive knee movement direction and velocities when supra-threshold SCS was active (Formento et al. 2018).

However, if we delivered supra-threshold short bursts of stimulation at the appropriate times while targeting specific roots, the natural firings in non-targeted roots were preserved even when SCS was active. Similarly, in the targeted roots natural information was fully preserved when SCS was not active. This approach allowed the sensory afferents to simultaneously carry action potentials generated by both the exogeneous electrical stimulation and natural proprioceptive information, thus creating an ecological or biomimetic neurostimulation protocol (Bensmaia 2015).

Far from being complete, this model provided important insights into the mechanisms of integration of peripheral inputs into propriospinal feedback circuitry. We applied these concepts to design a system that could implement this strategy and target large sensory fibers of specific roots to modulate spinal circuits promoting flexion and extension movements independently. We successfully tested these ideas first in monkeys (Capogrosso et al. 2016) and then in humans (Wagner et al. 2018). We think that our models that supported these translational developments can also provide a framework to interpret new human data (Angeli et al. 2018; Gill et al. 2018) and novel applications to different levels of the spinal cord (Barra et al. 2018).

This path, that goes through simulations, pre-clinical tests, and finally clinical trials, provides evidence that innovative neurostimulation therapies can be efficiently designed, optimized, and implemented when supported by computational tools.

### Other applications of computational modelling

There are several recent examples of successful application of computational modelling approaches to improve clinical neurostimulation technologies. One of the most powerful examples is in the field of deep brain stimulation (DBS). DBS is a common neurostimulation therapy used to treat movement disorders, such as essential tremor and Parkinson’s disease, and it is actively being investigated for additional indications, such as treatment resistant depression (Lozano et al. 2019). Computational models of DBS, largely driven by the rich history of computational modelling of SCS and peripheral nerve stimulation, have dramatically improved our scientific understanding of the direct neuromodulatory effects of DBS (Holt et al. 2016; McIntyre and Foutz 2013). During its original development, experimental studies suggested that DBS inhibited and/or decreased activity in the stimulated nucleus. However, computational models suggested that even though synaptic inhibition and/or depolarization blockade may occur at the soma, this suppression would have a limited effect on the output of neurons near the stimulating electrode because their axons can be directly excited by the DBS (McIntyre et al. 2004). This modelling data helped develop the hypothesis that DBS provides symptom relief by creating an informational lesion in the brain (Grill et al. 2004). Computational models of DBS have also been shown to provide an excellent tool for clinical decision support. Patient-specific models that account for patient anatomy and electrode locations have shown the ability to select stimulation parameters that provide superior clinical efficacy relative to stimulation parameters selected through standard clinical practice (Frankemolle et al. 2010). Computational models of DBS continue to be used as scientific and clinical tools and demonstrate how computational models can be used to transform a therapy. Furthermore, computational models are actively being utilized in several other neuromodulation therapies, such as vagus nerve stimulation, peripheral nerve stimulation, transcranial electrical stimulation, and transcranial magnetic stimulation (Aberra et al. 2020; Arle et al. 2016; Datta et al. 2016; Pelot et al. 2018; Shekhawat and Vanneste 2018).

In addition to providing a valuable scientific tool, computational models can also serve as a critical regulatory tool. Scientific evidence for medical device regulatory decision-making comes from four types of models: bench, animals, computers, and clinical trials (Morrison et al. 2018). Computational models have become an increasingly powerful tool for evaluating medical devices. In 2017, the United States FDA announced plans to integrate virtual testing and computational modelling into the medical device regulatory approval process to support faster, more efficient regulatory approvals without sacrificing patient safety or the confidence in regulatory decisions (Gottlieb 2017). Along with assessing device performance or safety, computational models are also increasingly being used within software platforms, serving as clinical decision support tools, and being embedded in medical devices. In a 2017 survey of scientists from the FDA’s Office of Science and Engineering Laboratories, approximately 44% of their medical device reviews included computational modelling in the submission (Morrison et al. 2018). The computational models typically provided simulation results as supporting evidence in a marketing application or the simulation was a medical device (e.g. clinical decision support). Therefore, computational models not only provide a means to advance our scientific understanding of neurostimulation technologies, but they may prove critical in transforming and accelerating the regulatory procedures for these devices.

### Artificial intelligence and models of neurostimulation

Increase in computational power over the last 40 years was critical to obtain precise volume conductor models of neural tissue (Capogrosso et al. 2013; Coburn and Sin 1985; Holsheimer 2002; Lempka et al. 2019; Rattay et al. 2000). Similarly, the increase in use and efficacy of artificial intelligence or machine learning will certainly open new possibilities with increased automatization. For example, it will increase our ability to automatically segment personalized patient data from magnetic resonance imaging data (Gaweł et al. 2018; Perone et al. 2018) and create three-dimensional models that are specific to each patient. This capability will open new realistic perspectives on the possibility to precisely characterize the voltage distribution for each patient, thus allowing a level of precision medicine that was unthinkable just a few years ago (Lempka et al. 2019).

### Model accuracy and validation

While we are arguing for a larger use of models in neuroscience, we must also acknowledge their limitations in neuroscience and neural engineering. In general, every theoretical model is only valid within a certain parameter range. For example, FEM solutions of neurostimulation are only valid within certain frequency ranges (Bossetti et al. 2008). Such constraints must be taken into account when choosing a specific model. On the other hand, the definition of these constraints is limited by our understanding of the specific neural system. Oddly enough, the act of modelling itself has a high scientific value in this regard. Indeed, one can only realize what information is missing while trying to model and reproduce the behaviour of a specific system. This process can and should be used to direct experimental research towards new directions that are necessary to complete our understanding. This directionality of scientific research is the prototype of the interplay between deductive and inductive scientific reasoning that is critical to advance human knowledge.

Finally, we would like to discuss the problem of model validation. Validating computational models in neuroscience and neurotechnology can be a daunting task. In fact, we believe that the perspective of model validation must be considered in the design phase to help determine the details that should be implemented in the models. Indeed, while an arbitrary level of detail can be included in neural models, it is important to keep in mind that the level of necessary “realism” is limited by the availability and accuracy of experimental data. For instance, in models of SCS for motor applications, a validation variable is the electromyographic activity induced in each muscle by SCS pulses (Capogrosso et al. 2013). In practice, this variable limits the details that can be implemented in computational models. While it would be possible to implement neuron models with complex morphologies and predict which neuron geometries are first recruited by SCS, it would not be possible to verify the model predictions using this experimental variable that represents a coarse approximation of the number of motoneurons recruited by SCS. Hence, every model must be designed from the start with a validation strategy in mind, and this validation strategy must guide and constrain the model assumptions and complexity. This approach will ensure that the models can be validated and can predict measurable experimental variables. This is true for both volume conductor models and models of complex neural networks.

### Our conclusion

We believe that computational models, can support a wide range of clinical neuromodulation therapies. Indeed, understanding how inputs to specific neural networks can be altered to modify aberrant behaviours of these networks can drive the definition of efficient neurostimulation therapies in diverse clinical applications. One thing that we must always keep in mind, is that whenever we apply electrical pulses to a given network in the nervous system, we are interfering with a living system that is relentlessly computing operations. The electrical stimulation will generate extrinsic inputs that will somehow interfere with these operations. If we can incorporate these computational principles into computational models, we could provide engineers and neuroscientists with powerful tools to understand how the nervous system responds to these technologies. Ultimately, we could provide the impatient information-era public community with hopes of accelerating the slow pace of clinical technology development and validation. At any rate, the growing use of computational models in neuroscience will eventually help normalize the philosophical approach to translational neuroscience, stepping away from purely experimental protocols towards a hypotheses-based verification process. Oh, and by the way, shouldn’t this be the way science is performed?

## Data Availability

Not applicable.

## Abbreviations

DBS: Deep brain stimulation
FDA: Food and Drug Administration
SCS: Spinal cord stimulation
